# *In vitro* Activity of Contezolid Against Methicillin-Resistant *Staphylococcus aureus*, Vancomycin-Resistant *Enterococcus*, and Strains With Linezolid Resistance Genes From China

**DOI:** 10.3389/fmicb.2021.729900

**Published:** 2021-08-19

**Authors:** Siheng Wang, Chang Cai, Yingbo Shen, Chengtao Sun, Qingxin Shi, Ningjun Wu, Shufang Zheng, Jiao Qian, Rong Zhang, Hongwei Zhou

**Affiliations:** ^1^Clinical Microbiology Laboratory, The Second Affiliated Hospital Zhejiang University School of Medicine, Hangzhou, China; ^2^China Australia Joint Laboratory for Animal Health Big Data Analytics, College of Animal Science and Technology, Zhejiang Agricultural and Forestry University, Hangzhou, China; ^3^Beijing Advanced Innovation Center for Food Nutrition and Human Health, College of Veterinary Medicine, China Agricultural University, Beijing, China; ^4^Clinical Laboratory Department, Taizhou Hospital of Zhejiang Province Affiliated to Wenzhou Medical University, Taizhou, China; ^5^Clinical Laboratory, Lishui People’s Hospital, Lishui, China; ^6^Department of Laboratory Medicine, Jinhua People’s Hospital, Jinhua, China

**Keywords:** contezolid, methicillin-resistant *Staphylococcus aureus*, vancomycin-resistant *Enterococcus*, linezolid, antibiotics, antimicrobial activity, multidrug-resistance, gram-positive

## Abstract

Contezolid is a novel oxazolidinone, which exhibits potent activity against gram-positive bacteria, including methicillin-resistant *Staphylococcus aureus* (MRSA), vancomycin-resistant *Enterococcus* (VRE), and penicillin-resistant *Streptococcus pneumoniae* (PRSP). In this study, the *in vitro* activity of contezolid was compared with linezolid (LZD), tigecycline (TGC), teicoplanin (TEC), vancomycin (VA), daptomycin (DAP), and florfenicol (FFC) against MRSA and VRE strains isolated from China. Contezolid revealed considerable activity against MRSA and VRE isolates with MIC_90_ values of 0.5 and 1.0 μg/mL, respectively. For VRE strains with different resistance genotypes, including vanA- and vanM-type strains, contezolid did not exhibit significantly differential antibacterial activity. Furthermore, the antimicrobial activity of contezolid is similar to or slightly better than that of linezolid against MRSA and VRE strains. Subsequently, the activity of contezolid was tested against strains carrying linezolid resistance genes, including *Staphylococcus capitis* carrying *cfr* gene and *Enterococcus faecalis* carrying *optrA* gene. The results showed that contezolid exhibited similar antimicrobial efficacy to linezolid against strains with linezolid resistance genes. In general, contezolid may have potential benefits to treat the infections caused by MRSA and VRE pathogens.

## Introduction

Increasing resistance to antibiotics in gram-positive cocci is a major concern of health care. In particular, the emergence of multidrug-resistant (MDR) bacteria leads to a decline in the treatment options. The World Health Organization (WHO) published a list of antibiotic-resistant “priority pathogens” in 2017 (Asokan et al., 2019). Among these pathogens, methicillin-resistant *Staphylococcus aureus* (MRSA) and vancomycin-resistant *Enterococcus* (VRE) are of particular concern since they are responsible for several severe infections. MRSA exhibits resistance to most available antibiotics, including fluoroquinolones and peptides, aminoglycosides, macrolides, and tetracycline (Osei Sekyere and Mensah, 2020). Therefore, novel antibacterial agents are urgently needed to treat infectious diseases caused by MDR gram-positive pathogens.

Oxazolidinones are a class of synthetic antimicrobial agents that are used to treat serious infections caused by gram-positive pathogens, including MRSA and VRE (Zurenko et al., 2001). Linezolid is the first member of the oxazolidinone antibiotics, which has some adverse effects (Hashemian et al., 2018). Clinical utilization of linezolid is restricted due to its toxicity such as myelosuppression and monoamine oxidase inhibition (MAOI) (Zahedi Bialvaei et al., 2017; Lee and Caffrey, 2018). In addition, the prevalence of linezolid resistance is increasing in many countries (Gu et al., 2013). The presence of *optrA* and *cfr* genes is one of the mechanisms mediating resistance to linezolid (Sadowy, 2018; Ruiz-Ripa et al., 2020).

Contezolid is a novel *ortho*-fluoro dihydropyridone oxazolidinone that replaces the morpholine in linezolid with a piperidinone (Meng et al., 2015). Contezolid inhibits the formation of functional 70S initiation complex by binding to the 23S rRNA region adjacent to the peptidyl transferase center of the 50S ribosomal subunit, thereby interfering with bacterial protein synthesis (Shinabarger, 1999). Contezolid has demonstrated potent antibacterial activity against resistant gram-positive pathogens (Gordeev and Yuan, 2014; Li et al., 2014; Wu et al., 2018). Additionally, contezolid showed antibacterial potential in multiple animal models, generally comparable with or slightly better than that for linezolid (Li et al., 2014), coupled with markedly attenuated human bone marrow cytotoxicity (Gordeev and Yuan, 2014; Huang et al., 2014; Li et al., 2014; Eckburg et al., 2017). In a phase III trial conducted in China (CTR20150855), contezolid was in development to treat complicated skin and soft tissue infections (Bassetti et al., 2020). According to the study, the most common adverse events associated with contezolid were gastrointestinal disorders such as nausea, and the incidence of myelosuppression was significantly lower than linezolid. Furthermore, contezolid displays a low propensity of spontaneous resistance (Gordeev and Yuan, 2014), and low potential to trigger resistance in *S. aureus* (Huang et al., 2014). Consequently, contezolid has the potential of offering a promising alternative therapy for MDR gram-positive organism infections.

The objective of this study was to evaluate the *in vitro* activity of contezolid relative to that of other comparator antimicrobial agents against MRSA, VRE, and strains carrying linezolid resistance genes using clinical isolates collected from China.

## Materials and Methods

### Bacterial Isolates

A total of 450 existing clinical isolates were collected from The Second Affiliated Hospital Zhejiang University School of Medicine, Huashan Hospital Affiliated to Fudan University, Henan Provincial People’s Hospital, and China Agricultural University from 2018 to 2020. The bacterial collection included 321 MRSA and 129 VRE isolates. Identification of strains was performed by matrix-assisted laser desorption ionization-time of flight mass spectrometry (MALDI-TOF/MS) (Bruker Daltonik, Bremen, Germany).

Kirby-Bauer method was used for MRSA and VRE screening according to the Clinical and Laboratory Standards Institute (CLSI) uniform standards. Isolates resistant to cefoxitin (8 μg/mL) with inhibition zone ≤21 mm were classified as MRSA and then confirmed by polymerase chain reaction (PCR) of *mecA* gene. Strains resistant to vancomycin with inhibition zone ≤14 mm were classified as VRE and then performed PCR of *vanA*, *vanB*, and *vanM* genes to determine vancomycin resistance genotypes. The *vanM* gene cluster sequences were determined by Sanger sequencing and BLAST program.

Eighteen previously described strains with linezolid resistance genes, including nine *Staphylococcus capitis* carrying *cfr* gene and nine *Enterococcus faecalis* carrying *optrA* gene collected from China Agricultural University (Wang et al., 2015) were used in this study. The primers used in this study were summarized in Table 1.

**TABLE 1:** Primers used in this study.

Primers	DNA sequence (5′–3′)	Length of target gene (bp)	References
*mecA*-F	AAAATCGATGGTAAAGGTTGGC	533 bp	Li et al., 2017
*mecA*-R	AGTTCTGCAGTACCGGATTTGC		
*vanA*-F	GGGAAAACGACAATTGC	732 bp	Dutka-Malen et al., 1995
*vanA*-R	GTACAATGCGGCCGTTA		
*vanB*-F	ATGGGAAGCCGATAGTC	635 bp	Dutka-Malen et al., 1995
*vanB*-R	GATTTCGTTCCTCGACC		
*vanM*-F	GTTTGGGGGTTGCTCAGAGG	1006 bp	Xu et al., 2010
*vanM*-R	TCACCCCTTTAACGCTAATACGATC		
*cfr*-F	TGAAGTATAAAGCAGGTTGGGAGTCA	746 bp	Wang et al., 2012
*cfr*-R	ACCATATAATTGACCACAAGCAGC		
*optrA*-F	AGGTGGTCAGCGAACTAA	1395 bp	Wang et al., 2015
*optrA*-R	ATCAACTGTTCCCATTCA		
			

### Antimicrobial Agents

Contezolid, linezolid, tigecycline, teicoplanin, vancomycin, daptomycin, cefoxitin, and florfenicol were obtained from National Institutes for Food and Drug Control. Broth microdilution panels were produced by Zhuhai DL Biotech Co., Ltd. The range of concentrations tested was: contezolid (0.125–16 μg/mL), linezolid (0.125–16 μg/mL), tigecycline (0.0625–2 μg/mL), teicoplanin (1–32 μg/mL), vancomycin (1–32 μg/mL), daptomycin (0.25–8 μg/mL), and florfenicol (1–32 μg/mL).

### Antimicrobial Susceptibility Testing

Antimicrobial susceptibility tests were performed by reference broth microdilution methods following CLSI procedures (CLSI, 2020a). Minimum inhibitory concentrations (MICs) were interpreted based on CLSI (CLSI, 2020b) and EUCAST.^1^ Quality control was conducted by using CLSI-recommended strains, including *S. aureus* ATCC 29213 and *E. faecalis* ATCC 29212. Statistical significance was calculated using the Chi-squared test via SPSS^®^ 20.0 software and *P* < 0.05 was considered as statistically significant.

## Results

### Antimicrobial Activity of Contezolid Against Tested MRSA and VRE Isolates

The MIC_50_ and MIC_90_ (MICs to inhibit the growth of 50% and 90% of organisms, respectively) of contezolid and comparator agents against MRSA and VRE strains were summarized in Table 2. Overall, contezolid demonstrated potent *in vitro* activity against MRSA and VRE isolates. All MRSA isolates tested were inhibited at a contezolid MIC value of ≤1 μg/mL (ranged from 0.25 to 1 μg/mL). Contezolid inhibited all VRE isolates at MIC ≤2 μg/mL (ranged from 0.25 to 2 μg/mL). Notably, only one of the VRE isolates showed a MIC at 2 μg/mL. MIC_90_ of contezolid against MRSA and VRE isolates were both ≤1 μg/mL. Moreover, there were 98.13% (315/321) of MRSA strains with MIC values ≤0.5 μg/mL and 79.84% (103/129) for VRE strains. In addition, for vanA- and vanM-type VRE strains, contezolid displayed similar MIC distributions, regardless of the vancomycin-resistant genotypes.

**TABLE 2:** *In vitro* activity of contezolid and comparator agents against MRSA and VRE strains.

Antimicrobial agent	MRSA		%S^a^	%R^a^	VRE		%S	%R
	MIC_50_ (μg/mL)	MIC_90_ (μg/mL)			MIC_50_ (μg/mL)	MIC_90_ (μg/mL)		
Contezolid	0.5	0.5	-^*b*^	-	0.5	1	-	-
Linezolid	0.5	0.5	100.0	0.0	0.5	1	100.0	0.0
Tigecycline	<0.0625	0.0625	100.0	0.0	<0.0625	<0.0625	100.0	0.0
Teicoplanin	<1	<1	100.0	0.0	8	32	80.6	19.4
Vancomycin	<1	<1	100.0	0.0	>32	>32	0.0	100.0
Daptomycin	0.5	0.5	100.0	0.0	2	4	100.0	0.0
Florfenicol	4	4	-	-	4	4	-	-

*^*a*^Criteria as published by CLSI and EUCAST. S, susceptible; R, resistant.^*b*^-, no breakpoint has been established.*

### Antimicrobial Effect of Contezolid Compared With Linezolid

Contezolid and linezolid displayed similar antimicrobial activity against MRSA and VRE isolates, with the same MIC_50_ and MIC_90_ values. However, when considering the MIC distributions, the number of strains with linezolid MIC values ≤0.5 μg/mL was less than that of contezolid in both MRSA and VRE isolates. Among the MRSA strains, there were 315 and 309 strains with MIC ≤0.5 μg/mL for contezolid and linezolid, respectively. However, it is worth noting that when it comes to VRE strains, there were 103 and 66 strains with MIC ≤0.5 μg/mL for contezolid and linezolid, respectively, which had statistical significance (*P* < 0.001) (Table 3).

**TABLE 3:** MIC distributions of two antimicrobial agents against VRE strains.

Antimicrobial agent	MIC distributions		
	≤0.5 μg/mL	1.0 μg/mL	2.0 μg/mL
Contezolid	103	25	1
Linezolid	66	63	0
*P* value	<0.001	-	-

Subsequently, the antimicrobial activity of contezolid was explored in strains carrying linezolid resistance genes. Both against *S. capitis* with *cfr* gene and *E. faecalis* with *optrA* gene, contezolid showed similar MIC distributions to linezolid (Table 4). These results demonstrated that contezolid displayed limited activity against strains carrying linezolid resistance genes.

**TABLE 4:** *In vitro* activity of contezolid and linezolid against strains with linezolid resistance genes.

Strains	Species	Drug-resistant genes	MIC (μg/mL)	
			Contezolid	Linezolid
103	*Staphylococcus capitis*	*cfr*	>16	>16
124	*Staphylococcus capitis*	*cfr*	>16	>16
127	*Staphylococcus capitis*	*cfr*	>16	>16
146	*Staphylococcus capitis*	*cfr*	>16	>16
161	*Staphylococcus capitis*	*cfr*	>16	>16
24	*Staphylococcus capitis*	*cfr*	>16	>16
390	*Staphylococcus capitis*	*cfr*	>16	>16
323	*Staphylococcus capitis*	*cfr*	>16	>16
283	*Staphylococcus capitis*	*cfr*	>16	>16
XY-22	*Enterococcus faecalis*	*optrA*	2	4
XY-29	*Enterococcus faecalis*	*optrA*	1	1
XY-11	*Enterococcus faecalis*	*optrA*	2	2
LY-4	*Enterococcus faecalis*	*optrA*	1	2
SS27	*Enterococcus faecalis*	*optrA*	2	4
JH2-2	*Enterococcus faecalis*	*optrA*	1	1
XY-12	*Enterococcus faecalis*	*optrA*	1	2
XY-9	*Enterococcus faecalis*	*optrA*	1	2
LY-9	*Enterococcus faecalis*	*optrA*	1	2

### Antimicrobial Effect of Contezolid Compared With Other Comparator Antimicrobial Agents

The MIC_50_ and MIC_90_ of contezolid against MRSA and VRE strains were not higher than that of teicoplanin, vancomycin, daptomycin, and florfenicol. However, the MICs of tigecycline were remarkably lower than that of contezolid against both MRSA and VRE strains. Accordingly, the antimicrobial activity of contezolid against MRSA and VRE isolates was similar to or slightly better than that of other comparator agents, except for tigecycline.

## Discussion

The antibacterial resistance toward currently available antibiotics is a widespread global health crisis. MDR gram-positive bacteria, accounting for both community-acquired and healthcare-associated infections, create numerous clinical challenges (Stevenson et al., 2005; Hoskins et al., 2018). Among them, MRSA and VRE deserve special attention for their high level of drug resistance. Accordingly, the development of new antibiotics is eagerly required to counter resistance.

Contezolid is a new oxazolidinone antibacterial agent with activity against gram-positive bacteria, including some multi-drug resistant organisms, such as MRSA, VRE, and PRSP (Gordeev and Yuan, 2014). Contezolid markedly reduces the potential for myelosuppression and monoamine oxidase inhibition compared to linezolid (Gordeev and Yuan, 2014), which seems to increase the clinical attractiveness of contezolid. Moreover, contezolid was reported to be not inferior to linezolid for the treatment of complicated skin and soft tissue infections with fewer hematology-associated adverse events in a phase three clinical trial conducted in China (Bassetti et al., 2020), indicating similar therapeutic outcomes between contezolid and linezolid. Contezolid acefosamil is the prodrug of the contezolid. *In vivo*, the double prodrug structure undergoes metabolic degradation including O-deacetylation and N-dephosphorylation, followed by the release of the active drug, contezolid. The prodrug form, which is water-soluble, could be used for either oral or intravenous administration of contezolid (Wang et al., 2021). Contezolid was approved for clinical use in China on July 2, 2021 for the treatment of complicated skin and soft tissue infections. And contezolid has been granted QIDP designation and Fast Track status by the US FDA.

In the present study, contezolid displayed potent activity against the whole collection of MRSA and VRE isolates. The antimicrobial activity of contezolid is comparable to that of linezolid based on MIC_50_ and MIC_90_ values. These results are in accordance with previous studies conducted in the United States and Europe (Carvalhaes et al., 2020). Notably, among VRE isolates, isolates with linezolid MIC values ≤0.5 μg/mL were statistically less than that of contezolid (*P* < 0.001). This indicated that the MIC distributions of contezolid against VRE are better than that of linezolid. Of concern, cross-resistance between linezolid and tedizolid, which both belong to oxazolidinone agents, was reported in *staphylococci* previously (Barber et al., 2016). In the current study, contezolid exhibited limited activity against strains with linezolid resistance genes. Consequently, the presence of the *cfr* and *optrA* genes may result in resistance to contezolid. This indicates that cross-resistance may also exist between contezolid and linezolid, which may limit the clinical application of contezolid. It also suggests the need to strengthen the clinical monitoring of cross-resistance between contezolid and linezolid. Among all the comparator agents tested, contezolid had relatively lower MIC_50_ and MIC_90_ values, indicating that its antimicrobial activity against MRSA and VRE was better than some antibiotics. Therefore, contezolid may offer another option for the clinical treatment of MDR gram-positive bacteria.

In summary, contezolid displayed potent *in vitro* activity against MRSA and VRE isolates collected from China. The antimicrobial activity of contezolid is similar to or slightly better than that of linezolid against MRSA and VRE isolates. However, cross-resistance may exist between contezolid and linezolid. The *in vitro* data in the current study imply that contezolid may be a promising candidate to treat MRSA and VRE infections, but may not be helpful for infections caused by linezolid-resistant strains. Further experimental and clinical researches are demanded to promote the progress of contezolid to reach clinical practice.

## Data Availability Statement

The original contributions presented in the study are included in the article/supplementary material, further inquiries can be directed to the corresponding author.

## Author Contributions

RZ and HZ designed the study. SW, YS, CS, QS, NW, SZ, and JQ did the experiment. CC, RZ, and HZ analyzed and interpreted the data. SW, HZ, and RZ wrote the manuscript. All authors read and approved the final manuscript.

## Conflict of Interest

The authors declare that the research was conducted in the absence of any commercial or financial relationships that could be construed as a potential conflict of interest.

## Publisher’s Note

All claims expressed in this article are solely those of the authors and do not necessarily represent those of their affiliated organizations, or those of the publisher, the editors and the reviewers. Any product that may be evaluated in this article, or claim that may be made by its manufacturer, is not guaranteed or endorsed by the publisher.

## Footnotes

1
https://eucast.org/clinical_breakpoints/
